# Sex‐related differences in corticospinal excitability outcome measures of the biceps brachii during a submaximal elbow flexor contraction

**DOI:** 10.14814/phy2.16102

**Published:** 2024-08-02

**Authors:** Olalekan B. Olarogba, Evan J. Lockyer, Angie K. Antolinez, Duane C. Button

**Affiliations:** ^1^ Human Neurophysiology Lab School of Human Kinetics and Recreation St. John's Newfoundland Canada; ^2^ Faculty of Medicine Memorial University of Newfoundland St. John's Newfoundland Canada

**Keywords:** motor‐evoked potential, transcranial magnetic stimulation, ultrasound

## Abstract

The purpose of this study was to investigate the effects of sex, muscle thickness, and subcutaneous fat thickness (SFT) on corticospinal excitability outcome measures of the biceps brachii. Eighteen participants (10 males and 8 females) completed this study. Ultrasound was used to assess biceps brachii muscle thickness and the overlying SFT. Transcranial magnetic stimulation (TMS) was used to determine corticospinal excitability by inducing motor‐evoked potentials (MEPs) at eight different TMS intensities from 90% to 160% of active motor threshold (AMT) from the biceps brachii during an isometric contraction of the elbow flexors at 10% of maximum voluntary contraction (MVC). Biceps brachii maximal compound muscle action potential (*M*_max_) was also recorded prior to and after TMS. Males had higher (*p* < 0.001) biceps brachii muscle thickness and lower SFT, produced higher levels of MVC force and had, on average, higher (*p* < 0.001) MEP amplitudes at lower (*p* < 0.05) percentages of maximal stimulator output than females during the 10% elbow flexion MVC. Multiple linear regression modeling revealed that sex was not associated with any of the neurophysiological parameters examined, while SFT showed a positive association with the stimulation intensity required at AMT (*p* = 0.035) and a negative association with biceps brachii pre‐stimulus electromyography (EMG) activity (*p* = 0.021). Additionally, there was a small positive association between muscle thickness and biceps brachii pre‐stimulus EMG activity (*p* = 0.049). Overall, this study suggests that some measures of corticospinal excitability may be different between the sexes and influenced by SFT and muscle thickness.

## Graphical abstract

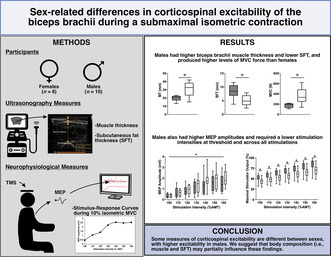

## 1 INTRODUCTION

The corticospinal pathway is vital for the execution of voluntary movements in humans (Chen, 2000). The excitability of the corticospinal tract can be assessed noninvasively by measuring motor‐evoked potentials (MEPs) elicited by transcranial magnetic stimulation (TMS) over the motor cortex (Chen, 2000). MEPs are recorded from the surface EMG from a target muscle and the size and shape of the MEP response (when normalized to the compound muscle action potential [*M*_max_]) reflects the net excitatory and inhibitory inputs to the corticospinal neurons and their axons (Chen, 2000; Lockyer et al., 2021). Several factors influence the excitability of the corticospinal pathway (for reviews see 21, 26), including voluntary muscle contraction, fatigue, muscle position, and state (active vs. rest), the task being performed, as well as age and exercise training status. More recently, biological sex has been proposed to influence corticospinal excitability, though the literature to date reports conflicting results. Some studies (Chagas et al., 2018; Inghilleri et al., 2004) have shown differences in corticospinal excitability between males and females, while others (Ansdell et al., 2019; Cantone et al., 2019; Keller et al., 2011; Pauhl et al., 2022; Pitcher et al., 2003; Yacyshyn & McNeil, 2020) report no differences. Other studies have shown that females have higher motor unit discharge rates (Harwood et al., 2014) and different motor unit recruitment strategies at lower intensity contraction efforts than males (Guo et al., 2022). Jenz et al. (2023)found that female spinal motor neurons where more excitable than males at a lower contraction intensity suggesting that descending motor commands may differ between the sexes. Some of these neural differences may be due sex hormones estradiol and progesterone (peak at much higher levels in females than males), which may alter monoaminergic output from the brainstem and overall excitability of the corticospinal tract (Rivas‐Grajales et al., 2023). Thus, it is plausible that biological sex may affect corticospinal outcome measures.

Body composition factors, such as muscle thickness and subcutaneous fat thickness (SFT) may also influence corticospinal excitability outcome measures. Males typically have larger muscle thickness (Abe et al., 2014; Frontera et al., 1991; Ichinose et al., 1998; Miller et al., 1993) and lower SFT (Bielemann et al., 2016; Perez‐Chirinos Buxade et al., 2018; Thiebaud et al., 2019) than females. However, the influence of these factors and sex‐dependent differences in these factors on corticospinal excitability outcome measures have yet to be considered in detail. Muscle thickness has long been implicated as a crucial determinant of force production and muscle strength (Close, 1972; Narici et al., 1989, 2016). Indeed, strong correlations exist between muscle thickness and muscle strength (Franchi et al., 2018; Miller et al., 1993), with larger muscles typically able to produce greater forces. While it is clear that muscle size is important for muscular strength, many other factors such as muscle architecture, muscle fiber contractile properties, and neural mechanisms also contribute to muscle force production (Narici et al., 2016). Given its role in the production of human movement, the corticospinal pathway has been implicated as a possible neural site involved in enhancing muscle strength (Kidgell & Pearce, 2011; Pearcey et al., 2014; Philpott et al., 2015). In particular, increases in muscle strength have been suggested to partially reflect the efficiency in descending neural drive to activate skeletal muscle (Kidgell & Pearce, 2011). Subcutaneous fat thickness is a measure of adipose tissue accumulation beneath the skin. Using neuromuscular electrical stimulation, larger SFT leads to higher stimulation intensities required to evoke responses (Doheny et al., 2010; Sakugawa et al., 2022). To date, however, no studies have directly investigated the influence of SFT on MEP amplitude. Given that the MEP is typically quantified from a target muscle using surface EMG, it is possible that larger SFTs would attenuate the evoked response due to the high resistivity of fat tissue (Doheny et al., 2010; Wagner, 2013).Whether SFT influences TMS‐evoked MEPs in a similar manner to electrical stimulation remains to be determined.

Therefore, the purpose of this study was to investigate if corticospinal excitability outcome measures are sex dependent and whether muscle thickness and SFT (assessed via ultrasonography) would influence these outcome measures in a sex‐specific manner. We hypothesized MEP amplitude would differ between sexes and that higher muscle thickness and lower SFT would be associated with larger MEP amplitudes. Given that males typically have larger muscle mass and smaller SFT than females, we hypothesized that MEP amplitudes would be larger in males.

## 2 MATERIALS AND METHODS

### 2.1 Participants

Based on previously published articles (Collins et al., 2017; Doheny et al., 2010; Thiebaud et al., 2019), a statistical power analysis calculation determined that 6 males and 6 females were required to ensure an alpha of 0.05 and a power of 0.8 for differences in muscle thickness and SFT. Eighteen healthy, recreationally active university student participants (10 males: 28.2 ± 7.6 years, height 178.6 ± 4.8 cm, weight 85.3 ± 13.3 kg and 8 females: 25.9 ± 5.2 years, height 160.6 ± 7.2 cm, weight 68.8 ± 15.6 kg) from Memorial University of Newfoundland volunteered to participate in this study. Participants were familiar with the stimulation techniques and isometric contractions used in the study. All participants received verbal explanation of the experimental protocol before written informed consent was obtained. Participants filled out a magnetic safety checklist (Rossi et al., 2011) and were also screened for any contraindications to exercise by completing the Physical Activity Readiness Questionnaire form (Bredin et al., 2013). Hand dominance (16 right‐hand dominance, 2 left‐hand dominance) was also determined using the Edinburgh handedness inventory (Veale, 2014), to ensure that experimental protocol was completed with the dominant arm. The sex of the participant was self‐reported. For female participants, the stage of the menstrual cycle and the use of oral contraceptives was not recorded. In addition, participants were also instructed to refrain from drinking caffeine for 2 h prior to the commencement of testing. All protocols were approved by the Interdisciplinary committee on Ethics in Human Research at Memorial University of Newfoundland (20220630‐HK). Additionally, the Tri‐Council guidelines in Canada were adhered to with the potential risks being fully disclosed to all participants.

### 2.2 Ultrasound measurements

Muscle thickness and SFT were measured from the dominant arm elbow flexors using a standardized ultrasonography procedure. Participants were seated in an armchair in a relaxed upright position with their feet distributed evenly on the floor. The elbow was bent at 90^0^ and the forearm rested on the armrest. Thickness measurements were taken at a standardized location of the halfway point between the acromion and olecranon on the anterior aspect of the upper arm over the midportion of the biceps brachii muscle belly.

**FIGURE 1:**
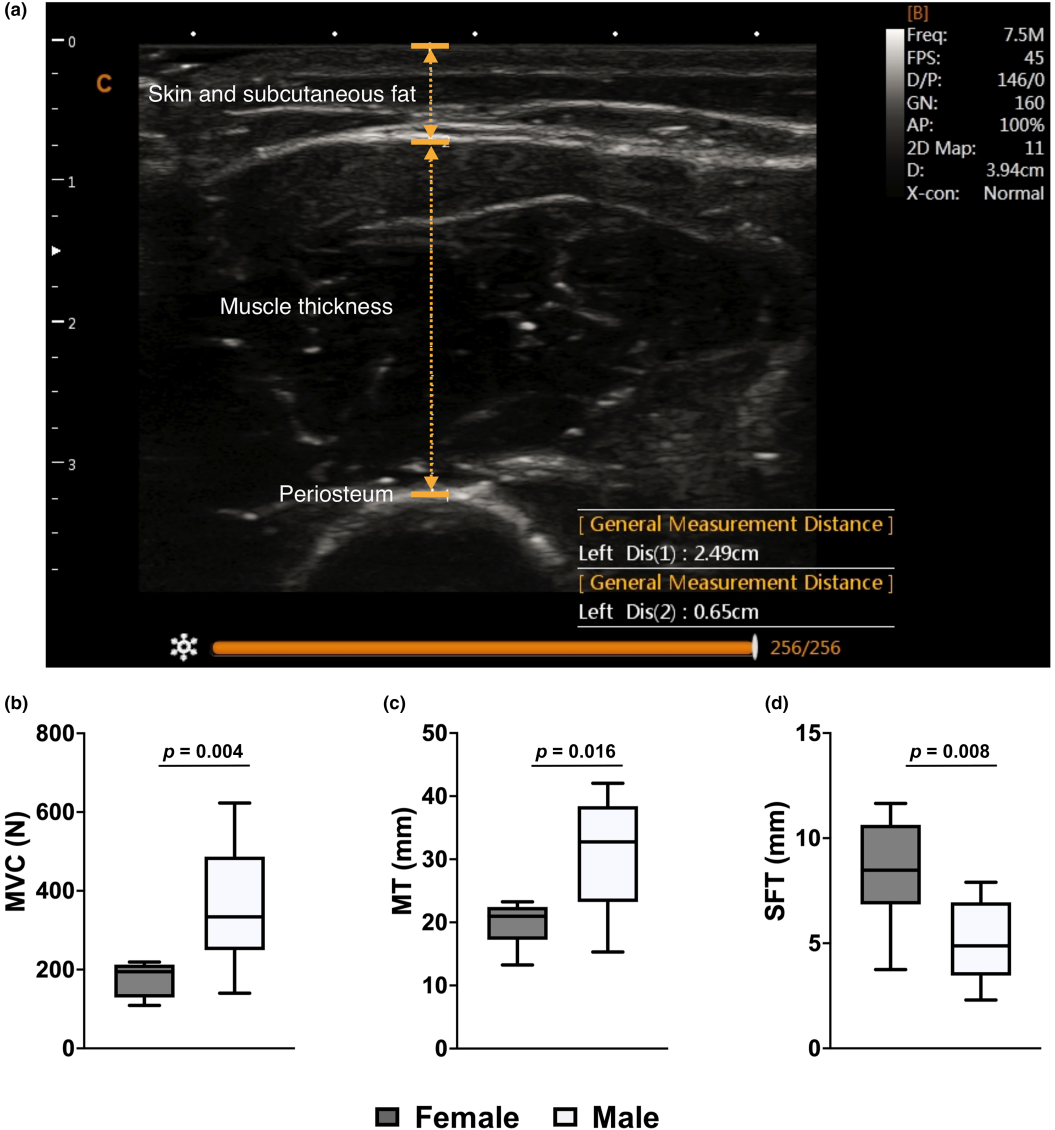
(a) Ultrasonography real‐time measurement of muscle thickness and SFT of biceps brachii from one ultrasound scan for one male participant. In this example, MT was measured to be 24.9 mm while SFT was measured to be 6.5 mm. Group data (*n* = 10 for males and *n* = 8 for females) for (b) MVC force, (c) bicep brachii MT, and (d) overlying biceps brachii SFT. Data presented as box and whisker plots. MVC, maximum voluntary contraction; MT, muscle thickness; SFT, subcutaneous fat thickness.

A Chison Sonobook 9 “portable” ultrasound system (Chison Sonobook 9, USA) was used in B‐mode with a linear array transducer (7.5 MHz, L12M‐T) to obtain all ultrasound images. Once the location of the mid‐muscle belly was identified, a thin film of transmissive gel was applied to the skin surface and the transducer was placed perpendicular to the skin/musculature to acquire a transverse image of the upper arm. The transducer was applied to the skin with just enough pressure to allow contact between its surface and the skin without compressing the subcutaneous fat layer. The transducer was moved slightly until image resolution was sufficiently clear for the subcutaneous fat/skeletal muscle interface and the skeletal muscle/bone interface to be simultaneously identified. Once a clear and suitable image was obtained, a still image was captured and the muscle thickness and SFT measurements were made. Muscle thickness was determined by measuring the linear distance on the image of the biceps brachii muscle from the edge of the subcutaneous tissue to the periosteum of the humerus. SFT was determined in a similar manner but included measuring the linear distance between the top of the fascia and bottom of the subcutaneous fat layer (Figure 1) (Sterczala et al., 2020). All scans were made by the same experimenter.

In addition to ultrasonography, SFT overlying the elbow flexors was also manually taken using Harpenden Skinfold Calipers (Baty International, West Sussex, England). With participants in the same position and using the same skin mark as used above for ultrasonography, the same experimenter recorded the biceps brachii skinfold measurements for each participant. Skinfold measurements were based on the Canadian Society for Exercise Physiology (Professional Fitness & Lifestyle Consultant Resource Manual, 1993). Briefly, two measurements were taken for each skinfold and if they were within 0.4 mm, the average was taken between the two. If the measurements were not within 0.4 mm of each other, a third measurement was taken and the average of the closest two measurements were taken.

### 2.3 Electromyography

EMG activity of the biceps brachii was recorded using 10 mm diameter MediTrace Pellet Ag/AgCl electrodes (disc shape, Covidien, Mansfield, USA). EMG was recorded using a bipolar configuration with an inter‐electrode distance of 20 mm (center‐to‐center) over the mid‐muscle belly of the participant's biceps brachii. The ground electrode was placed on the lateral epicondyle of the arm. Prior to the electrode placement, the participant's skin was prepared for all electrodes to reduce impedance and to obtain the best signal‐to‐noise ratio of EMG. The electrode site was shaved, abraded (using abrasive gel) to remove dead epithelial cells, and sanitized with isopropyl alcohol swabs. EMG was collected at 5 kHz using a CED 1401 interface and the associated Signal (version 5.11) software (Cambridge Electronic Design (CED) Ltd., Cambridge, UK). EMG signals were amplified (gain = 300) and filtered with a three‐pole Butterworth filter with cutoff frequencies of 10–1000 Hz with the CED 1902 amplifier.

### 2.4 Stimulation conditions

#### 2.4.1 Brachial plexus electrical stimulation (Erb's point stimulation)

Stimulation of the brachial plexus was used to measure participants' maximal compound motor unit action potential (*M*_max_). Erb's point was electrically stimulated via a cathode and anode (Meditrace Ag‐AgCl pellet electrode, disc‐shaped 10 mm diameter, Covidien, Mansfield, USA) positioned on the skin overlying the supraclavicular fossa and over the acromion process, respectively. Current pulses were delivered as a singlet using a constant‐current electrical stimulator (square wave pulse, 200 μs duration at 100–300 mA: model DS7AH, Digitimer Ltd, Welwyn Garden City, UK) during a sustained isometric contraction of the elbow flexors at 10% MVC. The electrical stimulation was gradually increased until the M‐wave of the biceps brachii no longer increased (Stefanelli et al., 2019). The stimulator setting used to evoke *M*_max_ was recorded and then increased by 10% to ensure maximal M‐wave throughout all trials. This stimulator intensity was used for the remainder of the experimental protocol. Two *M*_max_ trials were performed pre‐ and post‐TMS trials, with the average amplitude of the *M*_max_ recorded.

#### 2.4.2 Transcranial magnetic stimulation (TMS)

TMS was delivered using a Magstim 200 stimulator (Magstim, Whitland, Dyfed, UK). A circular coil (13.5 cm outside diameter) was positioned over the vertex of each participant's skull, with the direction of current flow in the coil preferentially activating the left or right motor cortex, depending on hand dominance. The vertex was located by measuring the midpoint between the participant's nasion and inion and the participant's tragus. The intersection of these two points was defined as the vertex and this point was clearly marked with a felt‐tipped dry‐erase marker. Electrical currents flowed in an anticlockwise direction through the circular coil and the induced current formed in the cortex flowed from anterior to posterior or vice versa to activate the right or left motor cortex (A side up for right limb dominance and B side up for left limb dominance) and subsequently activate the dominant biceps brachii. The coil was positioned directly over vertex and held firmly over the participant's skull tangential to the scalp by the same experimenter during each experimental session. Stimulation intensity started at approximately 30% maximal stimulator output and was increased gradually until active motor threshold (AMT) was determined. Participants were asked to hold a sustained isometric contraction of the elbow flexors at 10% MVC during AMT determination. AMT was defined as a clearly discernable MEP in the biceps brachii with an amplitude ≥100 μV in five out of ten stimulation trials. Once AMT was determined, eight experimental intensities (90%, 100%, 110%, 120%, 130%, 140%, 150%, and 160% of AMT) were calculated and were subsequently used to create a stimulus response curve for each participant. At each stimulation intensity, participants received 7 TMS pulses and the average MEP response was computed. This average MEP value at each intensity was subsequently used to create the stimulus response curve to indicate overall corticospinal excitability.

### 2.5 Experimental protocol

Participants completed a single experimental testing session lasting approximately 1.5 h. The procedure involved performing elbow flexion contractions at 10% of MVC while seated upright in a custom armchair (Collins et al., 2017; Stefanelli et al., 2019). The participants were first evaluated for anthropometric measures with which an ultrasound measurement for biceps brachii muscle size and ultrasound and skinfold measurement of SFT over the biceps brachii were performed. Participants were then prepped for EMG and the stimulation conditions. Participants performed submaximal isometric contractions for 5 s to get accustomed to producing force output. Participants then completed two elbow flexors MVCs, which were required to have force measurements (N) within 5% of one another to ensure maximal force output; if not, a third MVC was performed. The MVCs were then proceeded by a 10‐min rest period. After 10 min of rest, the intensities for each stimulation type were set. Corticospinal excitability (i.e., MEP) and peripheral excitability (i.e., *M*_max_) measurements were then taken from the biceps brachii during 10% MVC. The *M*_max_ was recorded before and after the TMS trials and the order of the stimulation intensity was randomized for each participant.

### 2.6 Data analysis

**FIGURE 2:**
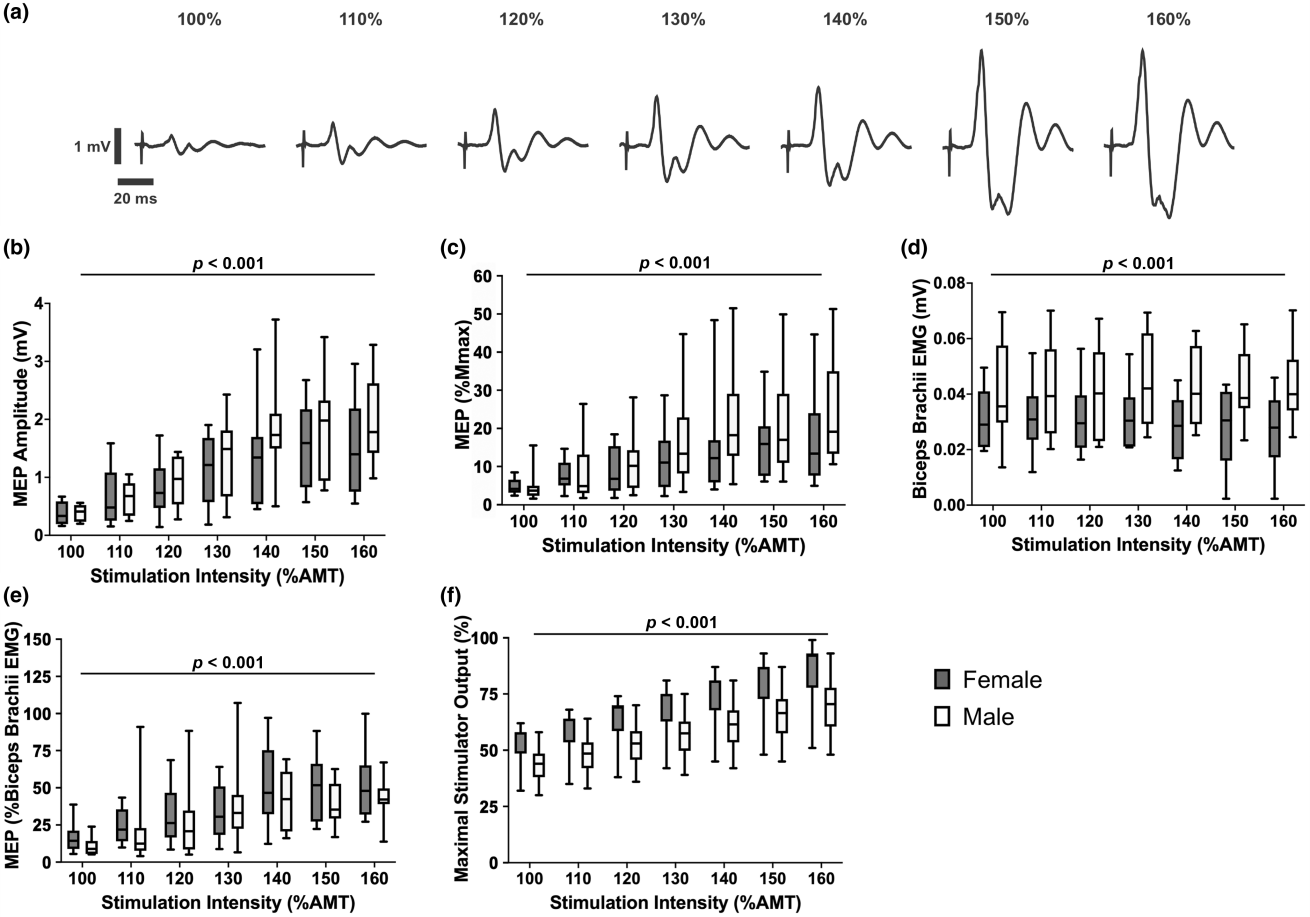
(a) Raw MEP responses from 100% to 160% of AMT during 10% MVC recorded from the biceps brachii of one male participant. In this example, the average *M*_max_ for this participant was 10.6 mV in amplitude. Group data (*n* = 10 for males and *n* = 8 for females) for (b) biceps brachii MEP amplitudes (in mV), (c) MEP amplitudes (% of *M*_max_), (d) biceps brachii EMG (mV), and (e) MEP/biceps brachii EMG ratios at stimulation intensities from 100% to 160% AMT during a 10% MVC, and (f) maximal stimulator output for TMS at each stimulation intensity. Data presented as box and whisker plots in (b–f) with significant main effects for sex reported. In (b and f), although not indicated, there was a significant increase in MEP amplitude (*p* < 0.02) as stimulation intensity increased from 100% to 140% AMT and maximal stimulator output % as stimulation intensity increased from 100% to 160% AMT. AMT, active motor threshold; EMG, electromyography; MEP, motor‐evoked potential; *M*_max_, maximal muscle compound action potential; MVC, maximum voluntary contraction.

Data for the muscle thickness and SFT were recorded in millimeters from the image produced using the B‐mode ultrasound. MVC force output was measured as the peak‐to‐peak amplitude from no force to maximum force. Prior to each MEP, the mean rectified pre‐stimulus EMG of the biceps brachii was measured for a 50‐ms time window. Corticospinal excitability (i.e., MEP) and peripheral excitability (*M*_max_) amplitudes were analyzed offline using Signal 5.11 software (CED, UK) after the experimental protocol was completed. Peak‐to‐peak amplitudes of evoked potentials (MEP and *M*_max_) were recorded from the initial deflection of the voltage trace to the return of the trace back to baseline EMG. To account for peripheral excitability, MEP amplitudes were also normalized to *M*_max_ and biceps brachii EMG. Stimulus response curves were generated by plotting the average of the seven raw MEP amplitudes across stimulation intensities (90%–160% threshold). The subsequent curves generated were then used to determine the area under the curve measurements. Figure 2a illustrates raw MEPs recorded from one participant across stimulation intensities (100%–160% of AMT). The area under the curve represents the summation of total corticospinal output over the range of TMS intensities used in the experiment (Iyer & Madhavan, 2019). The larger the value of the area under the curve, the greater the corticospinal output. The area under the curve for each stimulus response curve was obtained by trapezoidal integration of the curve's function using Prism 9 for MacOS (version 9.2.0; GraphPad Software LLC, CA, USA) (Nicolini et al., 2019).

### 2.7 Statistical analysis

Statistical analyses were computed using SPSS (SPSS 28.0 IBM Corporation, Armonk, New York, USA). Assumptions of normality (Shapiro–Wilk test) and sphericity (Mauchly's test) were tested for all dependent variables. If the assumption of sphericity was violated, the corrected value for non‐sphericity with Greenhouse–Geisser epsilon was reported. To examine the variability in MEP amplitudes and pre‐stimulus EMG for each stimulation intensity (100%–160% of AMT), a series of one‐way repeated measures ANOVA were computed. Separate two‐way mixed ANOVAs with between‐group factor of SEX (male and female) and repeated measures on within‐group factor of stimulation intensity (100%–160% of AMT) were computed for neurophysiological data. When significant interactions were found, paired *t*‐tests with the Bonferroni correction were performed. Partial eta squared (*η*_p_^2^) was used to determine the effect size of significant effects for ANOVAs (small: ≤ 0.06, medium: 0.07–0.14, and large: >0.14). To determine whether significant differences existed between pre‐ and post‐*M*_max_ amplitudes, a paired sample t‐test was performed. Additionally, independent t‐tests were also performed to assess whether a statistically significant mean difference existed in anthropometric, ultrasound, and stimulation data between sexes. For these analyses, Cohen's *d* (small: ≤0.2, medium: >0.2, and large: ≥0.8) effect sizes were calculated and reported. To examine the relationship between sex and anthropometric data on neurophysiology measures, multiple linear regression models were constructed on each neurophysiological variable (%maximal stimulator output at threshold, MEP amplitudes at 120% AMT, *M*_max_ amplitudes, stimulus response curve area under the curve values, and biceps brachii EMG, adjusting for sex, muscle thickness, and SFT). Upon checking the assumption of linearity *M*_max_ amplitudes and stimulus response curve area under the curve values were not linearly related, and as such were removed from the analyses. The regression coefficients are presented, including the 95% confidence intervals, along with the significance level of the main effects from the model (Table 2). All statistics were performed on group data and the statistical significance was set at *p* < 0.05. All data are reported as means ± standard deviation. Data in figures are presented as box and whisker plots.

## 3 RESULTS

Group data on anthropometrics, ultrasound, and force values for males and females are reported in Table 1. Overall, males were heavier (19%) and taller (10%), produced more elbow flexor force (106%; Figure 1b), had larger biceps brachii muscle thickness (35%; Figure 1c), and lower SFT (40%; Figure 1d) than females.

**TABLE 1:** Anthropometrics, ultrasound, force, and stimulation values for all participants, males, and females.

	All participants (*n* = 18)	Male (*n* = 10)	Female (*n* = 8)	*p*‐value	Cohen's *d*
Anthropometrics					
Age (years)	27.1 ± 6.5	28.2 ± 7.5	25.9 ± 5.2	0.23	0.3
Weight (kg)	88.0 ± 163	85.3 ± 13.3	68.8 ± 15.6	0.01*	1.2
Height (cm)	170.6 ± 10.9	178.6 ± 4.8	160.6 ± 7.2	< 0.001*	3.7
BMI (kg/m^2^)	26.7 ± 4.4	26.7 ± 3.4	26. 6 ± 5.5	0.49	0.01
Arm circum. (mm)	333.7 ± 42.2	347.7 ± 39.8	316.3 ± 40.8	0.06	0.8
Skinfold (mm)	8.1 ± 3.7	5.8 ± 1.8	11.0 ± 3.5	< 0.001*	2.8
Ultrasound assessment					
Fat thickness (mm)	6.6 ± 2.7	5.1 ± 1.9	8.5 ± 2.5	< 0.01*	1.8
Muscle size (mm)	25.9 ± 8.9	30.7 ± 9.2	19.9 ± 3.4	< 0.01*	1.2
Force					
MVC (N)	278.6 ± 149.5	361.5 ± 153	175.0 ± 43.8	< 0.01*	1.2

*Note*: Data presented as means ± standard deviation. *p* values are shown for sex differences (bold represents statistical difference).

Abbreviations: AMT, active motor threshold; AUC, area under the curve; *M*_max_, maximal muscle compound action potential; MVC, maximum voluntary contraction.

*Represents a statistical significance of *p* < 0.05.

### 3.1 Neurophysiological measures

#### 3.1.1 *M*_max_

Paired *t*‐tests revealed that *M*_max_ amplitudes were not different from pre‐TMS to post‐TMS protocol (9.8 ± 4.4 mV and 9.6 ± 4.7 mV, respectively; *t*_(16)_ = 1.52, *p* = 0.41, *d* = 0.03). Moreover, no sex differences in *M*_max_ amplitudes were found (males: 10.7 ± 4.1 mV and females: 8.5 ± 5.0 mV; *t*_(16)_ = 3.12, *p* = 0.32, *d* = 0.53).

#### 3.1.2 Consistency of MEP amplitudes and EMG

Results from the one‐way repeated measures ANOVA revealed that all MEP amplitudes were similar within AMT (*F*_(4.8,82.5)_ = 0.29, *p* = 0.91), 110% of AMT (*F*_(2.9,50.4)_ = 0.52, *p* = 0.67), 120% of AMT (*F*_(3.6,60.5)_ = 0.33, *p* = 0.83), 130% of AMT (*F*_(2.8,48.5)_ = 1.19, *p* = 0.32), 140% of AMT (*F*_(3.4,57.8)_ = 0.78, *p* = 0.51), 150% of AMT (*F*_(3.4,57.0)_ = 1.04, *p* = 0.38), and 160% of AMT (*F*_(3.7,62.8)_ = 2.19, *p* = 0.08). To determine if pre‐stimulus EMG from the biceps brachii prior to MEPs was different across trials, the average biceps brachii EMG was computed for each stimulation intensity (i.e., 100%–160% of AMT) and was then compared across intensities using a one‐way repeated measures ANOVA. Results revealed no differences (*F*_(6,119)_ = 0.11, *p* = 0.99) between any biceps brachii EMG data prior to each MEP during 10% MVC of the elbow flexors.

#### 3.1.3 Effect of sex on corticospinal excitability

Results from the two‐way mixed ANOVAs with between‐group factor of SEX (male and female) and within‐group factor of stimulation intensity (100%–160% of AMT) revealed main effects only for sex on raw MEP amplitudes (in mV), MEP amplitudes (% *M*_max_), biceps brachii EMG activity, and MEP/biceps brachii EMG ratio. Overall, males had higher MEP amplitudes (1.28 ± 0.63 vs. 1.05 ± 0.45 mV; *F*_(16,96)_ = 12.26, *p* < 0.001, *η*_p_^2^ = 0.67; Figure 2b), MEP amplitudes as a % of *M*_max_ (15.4 ± 7.4 vs. 11.7 ± 4.7; *F*_(16,96)_ = 10.31, *p* < 0.001, *η*_p_^2^ = 0.63; Figure 2c), biceps brachii EMG activity (0.042 ± 0.001 mV vs. 0.029 ± 0.001 mV; *F*_(16,96)_ = 46.31, *p* < 0.001, *η*_p_^2^ = 0.14; Figure 2d), lower MEP/biceps brachii EMG ratio (31.5 ± 14.4 vs. 37.1 ± 17.6; *F*_(16,96)_ = 6.83, *p* < 0.001, *η*_p_^2^ = 0.14; Figure 2e), and lower % maximal stimulator output used to elicit a MEP at each stimulation intensity (*F*_(16,96)_ = 251.4, *p* < 0.001, *η*_p_^2^ = 0.98; Figure 2f) compared to females, respectively. Although there were no interactions, Figure 2b–f includes data for each stimulation intensity for males and females. There were no differences in MEP stimulus response curve area under the curve values between sexes (males: 79.5 ± 31.7 and females: 65.8 ± 33.8; *t*_(16)_ = 0.89, *p* = 0.6, *d* = 0.39).

#### 3.1.4 Effect of stimulation intensity on corticospinal excitability

Results from the two‐way mixed ANOVAs with between‐group factor of SEX (male and female) and within‐group factor of stimulation intensity (100%–160% of AMT) showed a main effect of stimulation intensity on raw MEP amplitudes (*F*_(2.58,41.21)_ = 33.98, *p* = < 0.0001, *η*_p_^2^ = 0.67). Pairwise comparisons showed that mean MEP amplitudes increased (*p* < 0.02 for all comparisons) from 100% to 140% of AMT and then no longer increased (*p* > 0.9 for all comparisons) from 140% to 160% of AMT (Figure 2b).

### 3.2 Multiple regression analyses

Results of the multiple regression analysis are summarized in Table 2. Multiple linear regression models were constructed to determine the association between sex, biceps brachii muscle thickness, SFT, and neurophysiological measures. Specifically, separate multiple regression models were computed for each dependent neurophysiological parameter examined (i.e., % maximal stimulator output at threshold, MEP amplitudes at 120% AMT, and biceps brachii EMG), adjusting for sex, muscle thickness, and SFT. After adjusting for sex, muscle thickness, and SFT, the multiple linear regression model for each dependent variable demonstrated moderate to good fits to the data, with *R* ^2^ values ranging from 0.248 to 0.518. The results of the analysis revealed various associations between predictor variables and neurophysiological parameters examined. Specifically, SFT showed a positive association with the % maximal stimulator output required at threshold (*p* = 0.035) and a negative association with biceps brachii pre‐stimulus EMG activity (*p* = 0.021), suggesting that greater SFT was related to higher stimulation intensities to elicit threshold and lower EMG values, respectively. With regards to muscle thickness, there was a small positive association with biceps brachii pre‐stimulus EMG activity (*p* = 0.049), suggesting that larger muscle thickness was associated with slightly larger EMG activity in the biceps brachii. Sex did not show any associations with any of the dependent variables examined (*p* > 0.05 for all analyses).

**TABLE 2:** Results of multiple linear regression modeling on neurophysiological parameters.

	Dependent variables					
	%MSO at threshold		MEP amplitude at 120% AMT		Biceps brachii EMG	
	ß estimate (95% CI)	*p*‐value	ß estimate (95% CI)	*p*‐value	ß estimate (95% CI)	*p*‐value
Sex	−6.64 (−18.3 to 4.99)	0.241	−0.397 (−1.10 to 0.305)	0.245	−0.012 (−0.030 to 0.007)	0.194
MT	0.348 (−0.203 to 0.899)	0.197	−0.397 (−0.009 to 0.057)	0.146	0.0009 (6.03e‐006 to 0.002)	0.049*
SFT	1.99 (0.165–3.81)	0.035*	−0.076 (−0.186 to 0.034)	0.159	−0.003 (−0.006 to −0.0006)	0.021*
*R* ^2^	0.518		0.248		0.492	

*Note*: For all “sex” analyses on dependent variables, values are reported for males relative to females.

Abbreviations: AMT, active motor threshold; CI, confidence interval; EMG, electromyography; MEP, motor‐evoked potential; MSO, maximal stimulator output; MT, muscle thickness; SFT, subcutaneous fat thickness.

*Represents a statistical significance of *p* < 0.05.

## 4 DISCUSSION

This study explored whether or not there were sex‐dependent differences in corticospinal excitability outcome measures of the biceps brachii during 10% isometric contractions. Furthermore, we sought to determine if muscle thickness and SFT and the sex‐dependent differences in these two body composition factors altered the corticospinal excitability outcome measures. In line with previous reports (Abe et al., 2014; Bielemann et al., 2016; Frontera et al., 1991; Ichinose et al., 1998; Perez‐Chirinos Buxade et al., 2018; Thiebaud et al., 2019), we showed that males were larger in both height and weight, had larger muscle thickness and smaller SFT, and were able to produce more force than females. Coinciding with these differences in body composition and physical strength, males had higher MEP amplitudes (both raw and expressed as a % *M*_max_) at a lower percentage of maximal stimulator output compared to females. Moreover, males required a lower TMS intensity to obtain AMT compared to females. However, no differences in MEP stimulus–response curve area under the curve values were observed between the sexes. The multiple linear regression model did not reveal any associations between sex and the neurophysiological parameters when also adjusted for muscle thickness and SFT. Overall increased SFT was positively associated with increased stimulation intensity required to elicit AMT. Thus, the differences in the measures of corticospinal excitability between sexes are most likely not due to sex‐related differences in muscle thickness and/or SFT. Future studies should elaborate on these findings to further understand the effects of sex on corticospinal excitability.

### 4.1 Sex differences in muscle strength and body composition

It has long been established that males are generally stronger than females (Close, 1972; Ichinose et al., 1998; Laubach, 1976; Miller et al., 1993; Sale et al., 1987). This is especially true for muscles of the upper limb (Abe et al., 2014; Laubach, 1976) and for concentric contractions in particular (Porter et al., 1995; Singh & Karpovich, 1968). Sex differences in muscle strength likely arise due to a variety of biological and morphological factors, but muscle size is consistently cited as a primary contributor (Franchi et al., 2018; Ichinose et al., 1998; Narici et al., 2016; Nuzzo, 2023; Wust et al., 2008). Indeed, concomitant with greater muscle strength, males typically have larger muscles (i.e., larger muscle cross‐sectional area, volume, or thickness) than females (Miller et al., 1993; Nuzzo, 2023; Wust et al., 2008) and a greater proportion of force producing type II muscle fibers (Miller et al., 1993; Nuzzo, 2023; Sale et al., 1987; Wust et al., 2008). Additionally, male muscles also present with lesser infiltration of noncontractile tissue (i.e., fat) than females (Laubach, 1976). In the present study, males produced 106% greater MVC force than females during isometric contraction of the elbow flexors. Akin with previous suggestions (Abe et al., 2014; Franchi et al., 2018; Ichinose et al., 1998; Narici et al., 1989), we speculate that this observed sex difference in strength is at least partially due to differences in body composition and physical characteristics between females and males. In our study, ultrasonography measurements indicated that males had 35% larger biceps brachii muscle thickness (i.e., a measure of muscle size) and 40% less SFT overlying the biceps brachii than females. In addition, using skinfold calipers, females had a 47% larger biceps brachii skinfold thickness than males. Taken together, our findings provide further evidence to support the existence of sex differences in muscle strength and reiterate that muscle size may be a key mediator in these differences.

### 4.2 Sex differences in corticospinal excitability

Of the studies that have examined potential sex differences in corticospinal excitability, the majority support that corticospinal excitability is not different between sexes (Ansdell et al., 2019; Cantone et al., 2019; Keller et al., 2011; Pauhl et al., 2022; Pitcher et al., 2003; Yacyshyn & McNeil, 2020), while fewer suggest sex differences may exist (Chagas et al., 2018; Inghilleri et al., 2004). In the current study, we found that some corticospinal excitability outcome measures were different between females and males, while others were not. Notably, we found that MEP amplitudes were, on average, larger in males compared to females even though males required a lower stimulation intensity from TMS. Moreover, the minimum stimulation required to elicit a discernable MEP in the biceps brachii at AMT was higher in females than in males. Stimulus intensity to induce a MEP should be considered a factor of corticospinal excitability. Changes or differences in stimulus intensity to induce neuronal excitation is affected by the state (locomotion vs. non‐locomotion activity) (Power et al., 2010) and training status (exercise training vs. no training) (Beaumont & Gardiner, 2002; Lahouti et al., 2019; MacDonell et al., 2012) of the nervous system indicating a physiological change has occurred to or around the neurons. These findings therefore suggest that corticospinal excitability may indeed be sex dependent, with corticospinal excitability being higher in males than females. It is noteworthy to point out that the current findings were based on a similar relative contraction intensity (i.e. 10% MVC) which may not have occurred if the contraction intensity was matched absolutely between males and females. In contrast, the MEP stimulus–response curve area under the curve data revealed no differences between the sexes, suggesting that the summation of total corticospinal output was not different between males and females over the range of TMS intensities used. This finding is in line with previous work from Pitcher et al. (2003), who examined the effects of age and sex on MEP responses from the first dorsal interosseous (FDI) and reported that sex had a negligible influence (<3%) on the input–output characteristics of the corticospinal pathway (Pitcher et al., 2003). The present findings therefore suggest that differences in corticospinal excitability between the sexes might be TMS‐parameter specific. However, despite these findings, the multiple linear regression models revealed no relationship between sex and any of the neurophysiological parameters examined. Future work is certainly required to tease out potential mechanisms that may underlie these observations.

Although the multiple linear regression model did not reveal any associations between sex and the neurophysiological parameters when also adjusted for muscle thickness and SFT, we still believe that muscle thickness and especially SFT may play a role in the sex‐dependent differences in corticospinal excitability outcome measures reported here. Given that MEPs are recorded from the surface EMG signal, it is possible that the higher SFT overlying the biceps brachii in females acts as a buffer and therefore distorts the overall amplitude and detection of the MEP. This may be especially true at or near motor threshold, where slight deviations in SFT may impede the MEP signal and may result in a misidentification of the motor threshold. While this suggestion remains speculative, it is possible that the higher stimulation intensities required at AMT in females reflects the larger SFT overlying the biceps brachii. Indeed, evidence from studies using neuromuscular electrical stimulation suggests that higher amounts of fat tissue (due to its high resistivity) impair the conduction of current from the skin to the target neurons, thereby necessitating higher stimulus currents to evoke a response (Doheny et al., 2010; Wattananon et al., 2020). Although the mechanisms underlying responses to neuromuscular electrical stimulation and TMS of the motor cortex are quite different, we suggest that the larger SFT coupled with the smaller muscle thickness in females influenced the MEP responses observed presently. The present results therefore indicate that body composition factors, such as muscle thickness and SFT may be important factors to consider in future studies involving the assessment of the corticospinal pathway. Future research studies should recruit a variety of participants including male and female, trained and untrained, and of varying levels of weight categories (normal weight to obese) to get a better understanding of whether or not muscle thickness and SFT impact corticospinal excitability outcome measures.

### 4.3 Methodological considerations

Several factors must be considered in the interpretation of the current results. First, we did not monitor or record hormone levels for either sex in our study population. We did not control for fluctuations in ovarian hormones throughout the menstrual cycle, which have been shown to influence corticospinal excitability in female participants (Ansdell et al., 2019; Inghilleri et al., 2004). Thus, the current results of higher corticospinal excitability (i.e., higher MEP amplitudes and lower TMS stimulation intensities) in males compared to females during a 10% isometric contraction of the biceps brachii may not be translated to studies where ovarian hormones are monitored. The presence of estrogen and progesterone have shown to have net excitatory and inhibitory effects on nervous system excitability, respectively (Ansdell et al., 2019). Given that we did not monitor hormone levels, it is not possible to discern whether fluctuation in these hormone levels may underlie the present results. Similarly, we also did not monitor levels of testosterone in the study population, which has been shown to fluctuate throughout the day and can alter cortical excitability (Rivas‐Grajales et al., 2023; Zoghi et al., 2015). Thus, we have no way to know whether variations in testosterone within our study sample could be impacting the current findings. Future work should attempt to meticulously document these hormonal influences to determine whether they substantially impact data collected.

Second, all neurophysiological data (i.e., TMS‐evoked MEPs and EMG) were assessed during a 10% isometric contraction of the elbow flexors. It remains unknown whether the results would have been consistent between the sexes during higher intensity contractions, a different motor output (i.e., not an isometric contraction) or in muscles other than the biceps brachii. Indeed, corticospinal excitability has been shown to be modulated differently depending on the intensity of the contraction (Lockyer et al., 2019; Martin et al., 2006) and the motor output being performed (Kalmar, 2018; Lockyer et al., 2021) and in different muscles. Future studies should evaluate potential sex differences in corticospinal excitability during higher intensity contractions, during various motor outputs and different muscles to obtain a greater understanding of the relationships between body composition factors (i.e., muscle thickness and SFT) and corticospinal excitability outcome measures.

Third, in this study, TMS was used to produce MEPs, which provide indices of overall corticospinal excitability (Chen, 2000; Lockyer et al., 2021). Overall corticospinal excitability, however, is influenced by many factors, including factors at the supraspinal and spinal level (Chen, 2000; Lockyer et al., 2021). In previous studies from our lab examining the effects of chronic resistance training on corticospinal excitability, we gave electrical stimulation at the level of the cervicomedullary junction, referred to as transmastoid electrical stimulation, during various isometric contraction intensities to provide a measure of spinal excitability in addition to TMS‐MEPs (Pearcey et al., 2014; Philpott et al., 2015). By combining the two stimulation techniques, it allows for greater mechanistic insight underlying the change in overall corticospinal excitability. In the present study, however, we did not have a measure of spinal excitability and thus cannot rule out potential differences at the spinal motoneuron for the factors we examined. Indeed, a sexual dimorphism of motoneuron size in the spinal cord exists between males and females, with males having larger motoneuron size than females (Yuan et al., 2000). Furthermore, females have higher motor unit discharge rates (Harwood et al., 2014), different motor unit recruitment strategies, and motor neuron excitability levels (Jenz et al., 2023) at lower intensity contraction efforts than males (Guo et al., 2022). Thus, it is possible that spinal excitability might have also been different between the sexes in the present study. Future work should investigate whether differences in spinal excitability exist between the sexes and if so, should determine whether body composition factors, like muscle thickness and SFT, may be related to the differences.

Lastly, despite an a priori sample size calculation, the sample size in the present study is relatively small (males: *n* = 10; females: *n* = 8). Thus, the present results may not be extrapolated to a larger population. A study involving a large sample size may indeed show that there are relationships between muscle thickness and SFT and corticospinal excitability outcome measures.

## 5 CONCLUSION

The current results showed that corticospinal excitability projecting to the biceps brachii is, on average, higher in males than females, and that females require higher stimulation intensities to reach threshold compared to males. We sought to determine if body composition factors played a partial role in these sex‐dependent differences. Our findings did not indicate associations between sex and the neurophysiological parameters used to determine changes in corticospinal excitability when adjusted for muscle thickness and SFT. Overall, sex differences in corticospinal excitability outcome measures should be taken into consideration during experimental design in future studies.

## FUNDING INFORMATION

This research was funded by a Natural Science and Engineering Research Council of Canada Discovery Grant (201803876) to Duane Button.

## CONFLICT OF INTEREST STATEMENT

The author(s) declared no potential conflicts of interest with respect to the research, authorship, and/or publication of this article.

## ETHICS STATEMENT

All protocols were submitted to, and approved, by the Interdisciplinary committee on Ethics in Human Research at Memorial University of Newfoundland.

## Data Availability

The data that support the findings of this study are available from the corresponding author upon reasonable request.
